# Supplementary material for: Seasonal variations in respiratory morbidity in primary care and its correlation with the quality of air in urban Odisha, India
Source: PLOS Glob Public Health. 2024 Jan 29;4(1):e0002313. doi: 10.1371/journal.pgph.0002313 (PMC10824419; doi:10.1371/journal.pgph.0002313)
Supplement: Attachment — Submitted filename: CR PGPH.docx [file pgph.0002313.s002.docx]

**Reviewer #1**

Introduction:
The introduction can be improved upon. The following key areas are missing or not well-articulated.
What is the burden of respiratory diseases?

**Author’s response:** Data on burden of respiratory diseases in India added from both India as well as Odisha.

What is the burden of air pollution?

**Author’s response:** Data on burden of air pollution specifically average annual PM2.5 levels in India added.

What is the link between air pollution and respiratory diseases, which consequently prompted the authors to look for the trends in respiratory diseases and related air pollution levels?

**Author’s response:** We have already showed this in paragraph 2 of the introduction “PM2.5 is responsible for 59% of total deaths and DLAY…”; “air pollution causes and exacerbates chronic respiratory diseases such as…”

Where is the knowledge gap and how is the current study filling that gap?

**Author’s response:** This has been added in the last paragraph of the introduction section.

Line 121- pneumonia is not a chronic respiratory disease.

**Author’s response:** We are extremely sorry for this mistake. We have removed this.

Methods
The study sites were randomly selected. The authors need to clarify if are all the primary care facilities in the zones of the same level and do they see similar number of patients per month, otherwise, if the health facilities do not have similar background characteristics, there might be bias if a particular type is selected even if it was randomly.

**Author’s response:** Yes, in Indian healthcare system each primary care facility caters to the needs of 30,000 population thus, are similar in nature (average number of patients per month). We agree with the reviewer that there would have been bias in selecting the facilities and hence, we included only primary care facilities and not the other levels. We have added this in the methods section.

Line 156-157 states that data were collected from January to December in the 2018 and was 157 classified based on the major seasons according to local geo-climatic conditions. Was the classification done at data extraction or only information on month of clinic visit was captured and classification done at analysis?

**Author’s response:** The month-wise data was extracted and later classified at the time of data entry, we have now added this information in the manuscript.

The details on how information on air pollution was collected are missing. Did they for example consider the daily average or monthly average to decide on the PM2.5 levels for the different seasons?

**Author’s response:** We have now made a separate sub-section on air quality data and all relevant information has been added.

Data analysis

There is need to clarify on the denominator when calculating the incidence. The cases were derived from the clinic data base- so what was the reference population in this case? And why? In addition, data was collected for non-communicable diseases as well. When extracting data, did the authors consider only newly diagnosed patients or even those who had multiple visits in that year for re-fills/clinical review?

**Author’s response:** Since, our aim was to calculate the incidence of respiratory morbidities presenting at the selected facilities, we took the total number of cases (10900) present at the facilities as the denominator (population). This is now mentioned in the manuscript. Yes, we included only new cases and not the patients who came for clinical reviews again.

Ethical consideration
Line 181-182 states that authors did not have access to information that could identify participants. In most health systems, the names of the patients are recorded in the register. In this case, I assume that the data was extracted from such register. The authors need to clarify on the local systems on data capture from patients during a clinic visit, how the records are maintained, how they accessed them and how they ensured that all relevant data was accessed. Provide information on who did the data extraction. This information to be included in the description of the study sites and related practices in relation to the study.

**Author’s response:** Authors did not had access to information such as contact number and full address that could identify individual participants during or after data collection as the registers maintained by the clinician have name, age, sex, provisional diagnosis and treatment given.

Results
Provide detailed information on the air pollution levels during the study period, like monthly average, and show the trend.

**Author’s response:** Done as suggested.

The last paragraph in the results section indicates that there was some linkage PM2.5 and incidence of respiratory diagnoses. What type of linkage was this? And what does ‘positive’ mean? Given that the authors have details on diagnoses per season, and air pollution levels (as I assume), then they can analyze for correlation to enrich the study with such findings.

**Author’s response:** We have now done a test of significance i.e. Pearson Correlation that shows the data is significantly associated.

Discussion
In the second paragraph, the authors note that there was an increase in respiratory diagnoses between 2014 and 2018. Was this in any way related to air pollution levels?

**Author’s response:** This is a comparison between the burden of respiratory diseases only without taking into account air quality.

The authors make statements that are not supported by their results. For example, in paragraph 4, there is a statement ‘Furthermore, our findings show, respiratory illnesses increased with a rise in PM2.5 levels in air which also corresponds to the winter season’. Without tests of statistical significance, this statement cannot be validated. The same applies to other statements which mention the linkage without describing the type of linkage and how it was statistically arrived at.

**Author’s response:** We have now done a test of significance i.e. Pearson Correlation that shows the data is significantly associated. This makes these statements valid now.

The conclusion of a comprehensive action plan is also not backed by study results

**Author’s response:** We have deleted this.

We thank the reviewer for their valuable time and suggestions.

**Reviewer #2**

First, I would like to appreciate the efforts made by the authors for undertaking this research as it would add to the existing body of literature in the South Asian region on the topic of air quality and its impacts on health. However, the manuscript needs an overhaul before it can reach a publishable stage.


Major Comments:
• In general, the authors need to get the English corrected (sentence format, the sequencing and structure) by sending it to another colleague better in English. Even the authors can use various freely available websites that can support with English language editing.

**Author’s response:** Thank you for your suggestion. We have edited the manuscript for language and grammar. We hope it is up to the mark now.

• Significant concentrated efforts are required to modify the introduction as the essence and storyline is still missing but a better work can be done.

**Author’s response:** We followed all the suggestions by the reviewer as given below (in detail comments section). We also thank the reviewer for giving so much of time and efforts to help our manuscript strengthen.

• Are there any adjustments done for any other physical / physiological aspects of the patients concerned for performing this study, should be indicated.

**Author’s response:** No, we did not adjust for any other such factor, which we have now mentioned as a limitation of the study.

• Figures need special attention.

**Author’s response:** We have modified the figures by adding units and changing the layout.

General Comments:
Abstract:
1. The authors are suggested to rectify the abbreviation used PM2.5 / PM 2.5 should be written as “PM2.5”.

**Author’s response:** Thank you for your suggestion. We have now used PM2.5 throughout the manuscript.

2. Ln 87: This sentence should be shifted above “A positive linkage…….” To Ln 83. Such as the results should start from here and specifics should follow.

**Author’s response:** Done as suggested.

Introduction
3. Ln 123-127: They do not fit well in the introduction section, the authors can shift it to the study area, site description and general meteorology section. While describing the study area, the authors could refer previous studies done on the air quality of Bhubaneswar that indicates a seasonal pattern with a winter high and role of long-range transport, while a complete chemical, biological and physical profiling of wintertime particles could support the findings as well as defining the objectives of the current study.
• Seasonal progression of atmospheric particulate matter over an urban coastal region in peninsular India: Role of local meteorology and long-range transport
• Chemical, microstructural, and biological characterization of wintertime PM2.5 during a land campaign study in a coastal city of eastern India

**Author’s response:** We have shifted the lines 123-127 from introduction to study setting sub-section of methods. Additionally, as suggested by the reviewer, we have added details regarding air quality of Bhubaneswar and seasonal variation etc. whilst referring to the previous literature.

4. Ln 128-138: Needs more modifications in terms of the chronology followed for air pollution mitigation efforts. One major effort is the National Clean Air Program (NCAP) that is not mentioned. Further journal articles summarizing these efforts can also be thoroughly referred, while developing this section.

**Author’s response:** Thank you for the suggestion. We have now added all such measures in chronological sequence with reference.

5. The authors need to bring out the urgency of conducting this study i.e. Bhubaneswar is one of the most rapidly urbanizing cities in India and the projected population growth is also high. Hence, having a better understanding of the health effects would not only help the National efforts, but would be essential for framing state level efforts. While this example is for Bhubaneswar but sever such coastal cities in the South and South-east Asian region with similar or near similar challenges would also be benefitted from such a study.
• <https://moef.gov.in/wp-content/uploads/2019/05/NCAP_Report.pdf>
• National Clean Air Programme (NCAP) for Indian cities: Review and outlook of clean air action plans
• Monitoring particulate matter in India: recent trends and future outlook
Methodology

**Author’s response:** Introduction has been modified as suggested by the reviewer along with references.

6. Ln 156-160: A reference to the climatic classification presented in the study should be given.

**Author’s response:** Reference added.

7. The data collection section should be subdivided into two parts. First on health data collection and second on air quality data collection.

**Author’s response:** As suggested by the reviewer, the data collection sub-section has been divided into two parts.

8. One of the focus of this study is to understand the association of PM2.5 with health, making it essential to provide information on the same. The information should include what was the frequency of PM2.5 data used, how were they averaged or interpreted. From how many stations the data was used and what sort of an instrumentation was used. Something should be mentioned on the QA/QC protocols of collecting the data.

**Author’s response:** We have included data on number of stations, that the monthly average PM2.5 values were obtained, the process of using it in analysis i.e. hoe seasonal average was obtained. However, we do not have data on instrumentation used by Pollution control board.

9. Standard references for incidence of morbidity calculation should be used.

**Author’s response:** Reference added.

Results
10. Pg9 In the first paragraph, the “incidence increased from 53.11 per 1000 year during summer to 54.67 per 1000 person year during rainy season” is this s significant one?

**Author’s response:** Yes, this is significant as we have now added a Pearson Correlation value for it.

11. Supporting table one with certain descriptive statistics could have been better.

**Author’s response:** We agree with the reviewer that some statistics could have been better, however there are a lot of values with zero incidence which limits us in doing so.

12. Pg10 mentioned on the Pdf has a paragraph “During summer season, … PM2.5 levels.” Is a complete repetition of information provided in above paragraphs. Kindly refrain from doing so.

**Author’s response:** We have deleted this repetitive line.

13. There should be some more discussion keeping in view the figure 2 of this study, as this is the prime topic of the present manuscript.

**Author’s response:** We have added more lines in the result section pertinent to figure 2.

14. Even the figure 2 doesn’t provide any units

**Author’s response:** We have added units in the revised figure.

15. Statistical interpretations should be added to quantify the effect of PM2.5 and respiratory morbidities.

**Author’s response:** Thank you for the suggestion. We have added Pearson correlation and our findings suggest it to be correlated.

Discussions
16. Page no. 11 and 12 as mentioned on the pdf and its last paragraph and first paragraph respectively are extremely vague. Better to remove the same.

**Author’s response:** As suggested by the reviewer, we have removed the paragraphs.

17. The discussion in general should also be compared more in terms of the number where they are similar and dissimilar (both) and accordingly reach to a logical reasoning why such a result is seen at Bhubaneswar. To enhance the quality of study and put it in an international context, here studies done within the South and South-East Asian region should be compared and analysed.

**Author’s response:** Comparisons done as suggested.

We thank the reviewer for their valuable suggestions that have helped us improve the manuscript.
