# Supplementary material for: Seasonal variations in respiratory morbidity in primary care and its correlation with the quality of air in urban Odisha, India
Source: PLOS Glob Public Health. 2024 Jan 29;4(1):e0002313. doi: 10.1371/journal.pgph.0002313 (PMC10824419; doi:10.1371/journal.pgph.0002313)
Supplement: Attachment — Submitted filename: CR_Comments.docx [file pgph.0002313.s003.docx]

**Reviewer #1:**

Line 120-122: The statement about the burden of asthma and COPD better suited at the end of the next paragraph where information on disease burden is provided.

**Authors’ response:** We have shifted this statement to the end of next paragraph as suggested.

Line 171-174: the season that is being compared to the pre-monsoon is not stated.

**Authors’ response:** We have already mentioned this under the health data collection section.

Line 210-212: This information is about health data, but it has been put under the sub-heading of air pollution. This needs to be revised.

**Authors’ response:** Thank you for pointing this out. We have revised this as suggested.

In paragraph 4 of the discussions, the authors seem to authoritatively suggest a causal relationship between air pollution and respiratory morbidity as indicated in the sentence ‘It is evident that respiratory morbidities are evidently linked with the air pollution which needs attention at all levels’. However, it is important to note that this was a chart review where causal relationships are difficult to prove, and that there are many confounders of respiratory morbidity that were not controlled for. It might be better to use clear epidemiological terms like association or correlation throughout the documents rather than ‘linkage’ which is not very specific.

**Authors’ response:** We have replaced the term “linkages” with “correlation” throughout the manuscript including that in title.

We thank the reviewer for their valuable suggestions and time.

**Reviewer #2:**

The authors have made a decent attempt to address the comments. Some minor comments (appended below) need to be addressed before it can be published.
**Authors’ response:** We thank the reviewer for appreciating our efforts.

Comments
• Ln 200-212: Please mention the time of air quality data used. Something should be mentioned on the QA/QC protocols of collecting the data. Kindly talk to the PCB officials and mention it accordingly.

**Authors’ response:** We have added information on time of air quality data used along with QA/QC protocols.

• Figures 3 need special attention.

**Authors’ response:** We have again revised figure 3 and hope it is better suited now.

We thank the reviewer for their valuable suggestions and time.
